# The Stockholm Study: Over 30 years’ Observation of the Effect of Oral Infections on Systemic Health

**DOI:** 10.3390/dj10040068

**Published:** 2022-04-13

**Authors:** Jukka H. Meurman, Birgitta Söder

**Affiliations:** 1 Department of Oral and Maxillofacial Diseases, University of Helsinki, Helsinki University Hospital, PB 41, 00014 Helsinki, Finland; 2 Department of Dental Medicine, Karolinska Institute, 14104 Stockholm, Sweden; birgitta.soder@ki.se

**Keywords:** oral infection, systemic health, cohort study

## Abstract

The Stockholm Studies are a series of investigations started in 1985 and still ongoing. Out of 105,798 inhabitants, aged 30 and 40 years and living in the greater Stockholm area in Sweden, 3273 subjects were randomly selected. Of them, 1676 were clinically examined focusing on oral health. The subjects were then followed up using national population and health registers in order to study associations between oral health parameters and systemic health outcomes and finally death. The 35 years of observation provides unique possibilities to analyze, for example, how periodontitis links to a number of systemic health issues. The results have consequently provided numerous new associations and confirmed earlier observations on how poor oral health is associated with heart diseases and cancer.

## 1. Introduction

**Figure 1:**
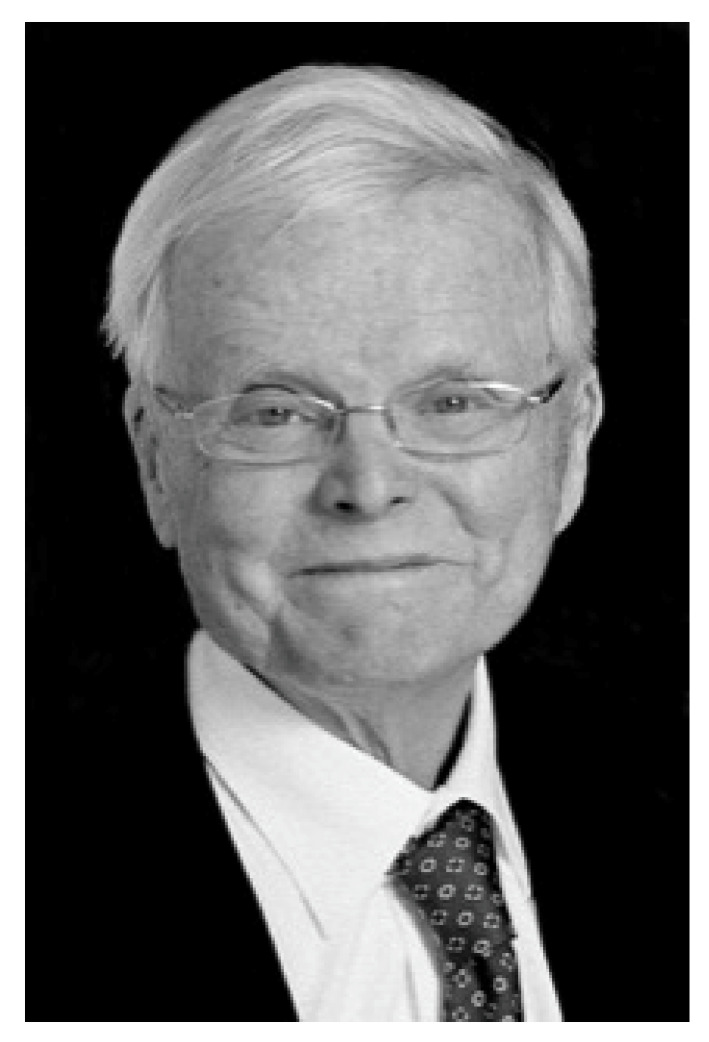
Professor Per-Östen Söder (1928–2020).

**Figure 2:**
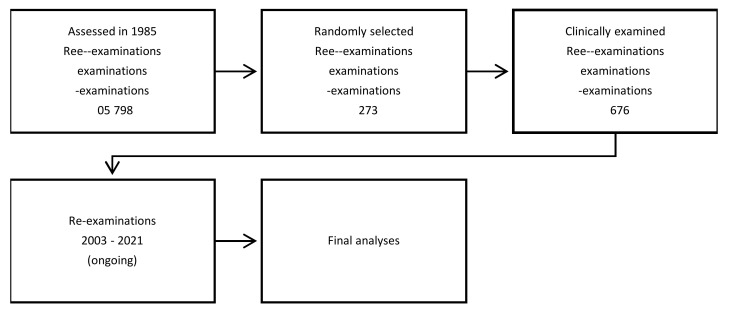
Profile of The Stockholm Study. At the re-examinations, the number of patients selected by computer program vary from 150–99.

In 1985, the late professor Per-Östen Söder started a cohort study at the Karolinska Institutet in Stockholm, Sweden (Figure 1). The subjects of the cohort were selected using the registry file of all of the inhabitants of the Stockholm metropolitan area and consisted of 3273 adults aged between 30 and 40 years and born on the 20th of any month between 1945 and 1954. This sample represented >100,000 inhabitants of the Stockholm area in the age group in question. In total, 1676 members of the cohort, 838 men and 838 women, agreed to participate and underwent an initial clinical dental examination in 1985–1986. The database is unique, now with a follow-up of over 30 years. Namely, in Sweden, all of the inhabitants have a unique code of identification and there are national cumulative population and disease registers. Thus, each subject´s life span can be followed up until death. In the series of studies described here, the following registers of the National Board of Health have been used in the analyses: social-economic register, open ward register, hospital register, dental treatment register, medication register, cancer register and death register (Table 1). Consequently, the disease burden of the subjects could be analyzed in view of their baseline oral health data. For example, the cumulative systemic disease burden of the patients with and without baseline periodontitis can now be followed up for more than 30 years through the registers. “Periodontitis” diagnosis in 1985 was based on measuring gingival pockets from 6 sites of each tooth to the nearest mm with a standard periodontal probe with pocket depth ≥5 mm used as unit in the analyses [1]. Outcomes such as prevalence of cardiovascular diseases or cancer at certain time points were observed during course of the investigation [2,3]. The following paragraphs present the results so far obtained from the Stockholm Study. The project is ongoing, and the profile of the cohort study is presented in Figure 2.

## 2. Studies on the Effect of Tobacco Use

The first studies from the cohort focused on the effect of smoking on periodontal health. As is well known, smoking is a marked risk factor for periodontitis, and this was indeed shown in the Stockholm Study. The results showed that self-reported smoking was significantly associated with deep periodontal pockets and missing teeth. For smokers, multivariate logistic regression analysis gave an odds ratio (OR) 3.21 with 95% confidence interval (CI) 1.73–5.74 of periodontal disease (*p* < 0.001). This association remained significant after adjustment for confounding factors (*p* < 0.01) [4]. Furthermore, the use of Swedish snuff, tobacco products with high nicotine content, was associated with poor periodontal health; however, it needs to be emphasized here that snuff users commonly smoke cigarettes [5]. In another study, life-long tobacco use, as measured by pack years of smoking, was found to link to unhealthy periodontal condition, particularly at the level of 15 pack-years or more. There was a significant positive association between current or former smoking and periodontal disease (OR 2.7, CI 1.7–4.3 and OR 2.0, CI 1.2–3.3, respectively) even after adjustment for dental plaque level. In this study, combining the life-long use of cigarette smoking and snuff use did not convey a decreased probability of being diagnosed with periodontal disease compared to exclusively smoking [4].

More recently, Julkunen-Iivari et al. [6] published results from the cohort where the aim had been to investigate if the use of tobacco products, that is smoking and snuff use, would be associated with reduced life span. It appeared that use of tobacco products was associated with lower education levels of the subjects (*p* < 0.001) as well as poor periodontal health (*p* < 0.001), aligning with earlier studies with the same cohort. However, contrary to expectation, tobacco users did not die earlier than non-users. Nevertheless, smoking is a significant risk factor for several systemic diseases, too, and must always be included in corresponding association analyses regarding oral health vs. systemic health.

## 3. Studies on the Effect of Periodontitis, Inflammatory Markers and Cardiovascular Diseases

Atherosclerosis is the pathology behind life threatening cardiovascular disease outcomes such as heart infarction and stroke. In the Stockholm Study project, an emphasis was placed on these important diseases prevalent in populations. First, a sample of 82 patients with periodontitis and 31 without periodontal disease were examined for oral health parameters, atherosclerosis and its risk factors. Carotid artery ultrasonography was performed where the common carotid artery intima-media thickness (IMT) and lumen diameter were measured, and the intima-media area (cIMA) calculated. The relationship between IMT and cIMA as dependent variables and periodontal disease, age, gender, body mass index, heredity for atherosclerosis, diabetes, hypertension, plasma cholesterol, smoking and education as independent variables, was analyzed using a multiple logistic regression model [2]. The result showed that the mean values of IMT and cIMA were significantly higher in patients with periodontal disease than in those without (*p* < 0.001 in both variables). The regression analysis identified periodontitis as a principal independent predictor of common carotid artery cIMA (OR 5.20; *p* <0.05) and IMT (OR 4.64; *p* < 0.05). It could thus be concluded that periodontal disease is associated with the development of early atherosclerotic carotid artery lesions [2]. The patients with periodontitis also had significantly higher total cholesterol (*p <* 0.01), low-density lipoprotein cholesterol (*p* < 0.05), and triglycerides (*p <* 0.01) than those without periodontitis. As discussed in more detail below, specific periodontal microorganisms seemed to induce a host response, reflected in increased concentrations of matrix metalloproteinase-8 and -9 (MMP-8 and MMP-9) in gingival pockets as well as in plasma, possibly triggering their up-regulation in blood [7]. These inflammatory markers indicate collagen degradation in tissue level [8] and have been used in studies investigating the associations between periodontitis and systemic health in general [9]. However, when discussing the role of MMPs in general regarding connection with periodontitis, gender differences and smoking habits also need to be taken into account. Virtanen et al. [10] showed in the Stockholm Study material that MMP-13 may have gender implications in periodontitis.

The reason why infections such as oral infections associate with cardiovascular diseases are thought to be the subsequent chronic and often subclinical inflammation that, in turn, triggers pathogenic alterations in the intima of blood vessels leading to lipid and mineral accumulation and then to atheroma formation. C-reactive protein (CRP) is a known serum marker of inflammation, and it has been shown to be associated with atherosclerosis [11]. In the Stockholm Study, however, in the patients examined with carotid artery ultrasonography, this association could not be found. The cIMA and IMT did link to periodontitis as said above, but neither of these variables showed association with CRP values [12,13]. However, as stated above, other elevated inflammatory markers were nevertheless found in the patients with atherosclerosis and periodontitis. Periodontitis was found to predict increased MMP-9 and tissue inhibitor of matrix metalloproteinases (TIMP-1) and their ratio MMP-9/TIMP-1. These values were indeed significantly higher in plasma from subjects with periodontal disease and atherosclerosis when compared with healthy subjects (OR 2.58, 5.53 and 3.41, respectively). Classical atherosclerosis risk factors, such as increased total cholesterol, age, and sex (female), were significant predictors in the model discussed here [14].

Correspondingly, other inflammatory markers such as leukotriene B_4_ and cysteinyl-leukotrienes were detected in gingival crevicular fluid (GCF) from subjects with a high dental plaque index (PLI > 0.3), supporting an increased leukotriene formation in periodontitis [15]. Patients with atherosclerotic plaques had significantly elevated concentrations of cysteinyl-leukotrienes in their GCF when compared with those without visible dental plaque. Thus, the results suggest that increased leukotriene formation may also represent a possible link between periodontitis and atherosclerosis and might be used as risk factor marker for the diseases [15,16].

## 4. Studies on the Role of Oral Microorganisms and Poor Oral Health

Obviously, oral microorganisms detected in patients with periodontitis have been of interest in studies regarding the link between oral infections and cardiovascular diseases [17]. In The Stockholm Study, the Söder group has investigated the prevalence of certain periodontal microorganisms in this perspective. GCF samples have been taken to analyze the bacteria Aggregatibacter actinomycetemcomitans, Porphyromonas gingivalis, Prevotella intermedia, Prevotella nigrescens, Tannerella forsythia and Treponema denticola. The prevalence of these bacteria was then statistically analyzed with respect to a number of oral health variables, atherosclerotic risk factors, results from serum analyses, and findings at the carotid ultrasonic examination. The results showed significant differences in the detection of *P. gingivalis* (*p* < 0.001), *P. intermedia* (*p* < 0.01), P. nigrescens (*p* < 0.001) and *T. forsythia* (*p* < 0.001), but also in the levels of MMP-8 and MMP-9 in GCF between patients with and without periodontitis. *T. forsythia* (OR 10.1; *p* < 0.001) and age (OR 5.54; *p* < 0.01) appeared to be the main independent predictors for high MMP-8 in GCF [7].

In later studies of the same patients, significantly higher total cholesterol (*p* < 0.01), low-density lipoprotein cholesterol (*p* < 0.05), and triglycerides (*p* < 0.01) were observed in patients with than without periodontitis. Further, smoking (OR 5.64; *p* < 0.001), level of education (OR 5.02; *p* < 0.05) and the presence of *P. gingivalis* (OR 6.50; *p* < 0.05) were associated with periodontitis. Explanatory factors for the increased cIMA were periodontitis (OR 4.22; *p* < 0.05), hypertension (OR 4.81; *p* < 0.05), high body mass index (OR 5.78; *p* < 0.01), male gender (OR 3.30; *p* < 0.05) and poor socioeconomic status (OR 4.34; *p* < 0.05). *P. nigrescens* (OR 4.08; *p* < 0.05) and *P. gingivalis* (OR 7.63; *p* < 0.01) also appeared as explanatory variables associated with increased cIMA values in this series [18]. The results indicate an association of periodontitis with several atherosclerotic risk parameters.

In further studies, a sample of 99 patients from the cohort were clinically re-examined and salivary and GCF samples taken for analyses of salivary albumin, total protein, immunoglobulins A, G and M and MMP-8. The same periodontal microorganisms as above were detected using PCR. It appeared that periodontitis patients were more often infected by *P. gingivalis* (*p* < 0.05), *P. intermedia* and *T. denticola* (*p* = 0.01) than non-periodontitis patients. Salivary albumin and protein concentrations were significantly higher in patients infected with *T. denticola* (*p* < 0.05) while MMP-8 levels were significantly higher in those with *T. denticola* (*p* < 0.001) and *T. forsythia* (*p* < 0.01). However, no difference between groups was found in salivary immunoglobulin concentrations [12]. Thus, infection with specific periodontal microorganisms reflected in salivary albumin concentration which is a marker of oral epithelial barrier while MMP-8 indicates collagen breakdown [19,20].

Of the 1676 clinically examined participants, 39 subjects (2.3%) had been diagnosed with a stroke during 26 years of follow-up. Those who had suffered a stroke had smoked more when calculated the pack-years (*p* < 0.01). But the most interesting finding was related to gingivitis. A high GI score was significantly associated with the incidence of stroke with OR 2.20 (CI 1.02–4.74) [21]. Furthermore, high dental calculus score was found to associate with angina pectoris in this study with OR 2.21 (CI 1.17–4.17; *p* < 0.05) [22]. In the multiple regression analyses, mortality was the outcome of the study and the results showed that a high dental calculus score associated with death from heart infarction with an OR 2.30 (CI 1.05–5.06). The analyses were controlled for age, gender, dental visits, dental plaque, periodontal pockets, education, income, socioeconomic status, and pack-years of smoking [23].

Earlier analyses of the Stockholm Study cohort showed that poor oral health parameters were associated with premature death of the subjects. Young individuals with periodontitis and missing molars seemed to be at increased risk for premature death by life-threatening diseases, such as neoplasms and diseases of the circulatory and digestive systems [24]. Missing molars indicate earlier dental infections because they are seldom extracted unless there is dental pathology involved.

Finally, in addition to gingivitis, derived periodontal disease also apical periodontitis links to cardiovascular diseases. In The Stockholm Study, regression analyses controlled for age, gender, income, smoking and periodontitis, showed apical periodontitis to associate with cardiovascular disease with OR 3.83 (CI 1.18–12.40; *p* < 0.05). These results were from a subsample of 120 patients in that had been radiographically examined for dental pathologies [25].

## 5. Studies on Oral Infections and Cancer

The history of the role of infections in the etiology of malignancies goes back to the year 1911 when American pathologist Peyton Rous detected a virus that caused sarcoma in chicken. This was then verified to be true as late as 1975, even though Rous had received a Nobel Prize for his detection already in 1966. In 1980s, German researcher Harald zur Hausen found out that certain human papilloma viruses can cause cervical cancer. He received the Nobel Prize for these studies in 2008. Further in the 1980s, when Helicobacter pylori was detected by Australian Nobel laureates Barry Marshall and Robin Warren, who received their Prize in 2005, the door was open to investigate the role of also bacterial infections in carcinogenesis [26]. This introduction shows how significant the role of infections is in carcinogenesis, or in malignant transformation in general and that the landmark studies in the area have all been recognized by the Nobel committee.

The Söder group became interested in investigating the role of oral infections in cancer. This development was natural, taking into account the high prevalence of dental infections in populations and the fact that cancer is the second most important cause of death after cardiovascular diseases. Infection-driven inflammations have been estimated to be involved in the pathogenesis of approximately 15–20% of human malignancies [27]. Hence, inflammation caused by infections might be one of the most important preventable causes of cancer and thus controlling the role of oral microbiota is emphasized in this regard [28].

The Stockholm Study provided excellent data for analyzing the associations between poor oral health and malignancies. First, data on breast cancer were published and the results showed that the incidence of breast cancer was 1.75% in subjects who had periodontal disease and/or any missing molars, and 0 in subjects who had periodontal disease but had no missing molars. Of the subjects with periodontal disease and any missing molar in the mandible 5.5% had breast cancer in comparison to 0.5% of the subjects who had periodontal disease but no missing molars (OR 2.36 CI 1.07–5.21; *p* < 0.02) [29] .

Further investigations showed that poor oral health was also associated with cancer-caused mortality. High dental plaque index appeared to associate with 1.79 times the OR of death (CI 1.01–3.19; *p* < 0.05). Age increased the risk with an OR of 1.98 (CI 1.11–3.54; *p* < 0.05) and male gender with an OR of 1.91 (CI 1.05–3.46; *p* < 0.05). The malignancies were more widely scattered in men, while breast cancer was the most frequent cause of death in women [3]. In a 26-year follow-up data of The Stockholm Study cohort, gingival inflammation as assessed by a high GI score appeared to link to cancer in general. As expected, pack-years of smoking was a principal independent predictor with OR 1.32 (CI 1.05–1.67) while gingival inflammation gave an OR 1.29 (1.00–1.65), respectively, in multiple logistic regression analysis with cancer as the dependent variable and several independent variables in this cohort [30].

When studying the incidence of cancer during the long observation time in the database, it appeared that of the 1390 subjects who underwent clinical oral examination in 1985, cancer had been registered in 71 patients by year 2009. In this material, similarly to the previous study, missing molars associated with cancer with OR 2.62 (CI 1.18–5.78). For comparison, the OR for age was 1.91 (CI 1.06–3.43), respectively [31].

Finally, closer to the etiological agents earlier discussed, a study was created with the hypothesis that certain periodontal microorganisms associate with malignancies. A sample of 99 clinically examined patients of The Stockholm Study cohort was used to investigate the associations between harboring periodontal microorganisms *A. actinomycetemcomitans*, *P. gingivalis*, *P. intermedia*, *T. forsythia* and *T. denticola*. Here, similar to our earlier study, gingival inflammation emerged statistically as the strongest sign of inflammation (Eigen value 4.11 and Explained Variance 68.44%) in the 2008–2016 Swedish National Cancer Register used. Of the bacteria analyzed, *A. actinomycetemcomitans* showed strong association with malignancy in 32 out of the 99 patients while *P. gingivalis* and *P. intermedia* were more prevalent among patients without malignancy. In principal component analyses, *A. actinomycetemcomitans* was in the strongest component while the second strongest component consisted of a combination of *T. forsythia* and *T. denticola*, respectively [32]. Thus, certain periodontal pathogens indeed seemed to associate with malignancy, but no causality can be drawn from this kind of small-scale investigation.

Taken together, cancer data from The Stockholm Study indicate that oral infections may indeed have a role in carcinogenesis. The putative pathogenic mechanisms that explain the associations detected are similar to those with cardiovascular diseases. Namely, the common nominator is the chronic and often subclinical inflammation caused by oral microorganisms, leading to systemic consequences. Malignant transformation in the cellular level is then one of them [33].

## 6. Oral Infections and Autoimmune Diseases

Immunological mechanisms are known to affect the oral cavity with characteristic pathologic alterations [34]. Infections in general have been suggested to trigger autoimmune diseases such as rheumatic diseases and diabetes and their oral microorganisms such as *P. gingivalis* may play a role [35]. In The Stockholm Study, the prevalence of autoimmune diseases was investigated and 50 such patients were detected in the database. In more detail, the result showed that poor oral hygiene as assessed with high plaque index score was significantly higher among the patients (70%) compared with the subjects with no autoimmune disease (54%, *p* < 0.05). In this data, however, no statistically significant difference between the groups was found in any other dental index registered [36]. Nevertheless, the result showed that subjects with poor oral hygiene were more likely to develop autoimmune diseases in 30 years.

## 7. Conclusions

The studies cited above emphasize the importance of collecting long-term register data where both the oral health parameters and systemic health parameters can be extracted. The Stockholm Study databases are in many regards unique in this respect and, as the data accumulates all the time. Hence, future analyses with this material are expected to bring out many disease associations where poor oral health can be expected to associate. The results published so far have clearly shown the importance of maintaining good oral hygiene through lifetime. Diagnosing and treating dental and oral inflammations at the individual level cannot be overemphasized. However, all the results cited in the previous paragraphs only show statistical associations. Therefore, one must keep in mind that no conclusions of causalities can be drawn from these kinds of investigations.

## Figures and Tables

**Table 1:** Register databases used in the series of studies.

Database	Source
Socio-economic population register	Statistics Sweden (SCB)
Open ward (polyclinic) register	Swedish National Board of Health and Welfare
Hospital register	Swedish National Board of Health and Welfare
Dental treatment register	Swedish National Board of Health and Welfare
Prescription medicine register	Swedish National Board of Health and Welfare
Heart infarction register	Swedish National Board of Health and Welfare
Cancer register	Swedish National Board of Health and Welfare
Death register	Swedish National Board of Health and Welfare
